# Health service experiences and preferences of frail home care clients and their family and friend caregivers during the COVID-19 pandemic

**DOI:** 10.1186/s13104-021-05686-6

**Published:** 2021-07-14

**Authors:** Lori E. Weeks, Sue Nesto, Bradley Hiebert, Grace Warner, Wendy Luciano, Kathleen Ledoux, Lorie Donelle

**Affiliations:** 1 School of Nursing, Dalhousie University, Halifax, NS B3H 4R2 Canada; 2 School of Occupational Therapy, Dalhousie University, Halifax, NS B3H 4R2 Canada; 3 Arthur Labatt School of Nursing, Western University, London, ON Canada

**Keywords:** COVID-19, Remote monitoring, Home care, Older adults, Frailty

## Abstract

**Objective:**

The COVID-19 pandemic has brought about a major upheaval in the lives of older adults and their family/friend caregivers, including those utilizing home care services. In this article, we focus on results from a qualitative component added to a pragmatic randomized controlled trial that focuses on the experiences of our study participants during COVID-19. A total of 29 participants responded to the COVID-19 related questions focused on their health services experiences and preferences from March-June 2020 including 10 home care clients and 19 family/friend caregivers in the provinces of Ontario and Nova Scotia, Canada.

**Results:**

Many participants were affected drastically by the elimination or reduction of access to services, highlighting the vulnerability of home care clients and their caregivers during COVID-19. This took an emotional toll on home care clients and increased the need for family/friend caregiver support. While many participants expressed reduced desire to utilize residential long-term care homes, some caregivers found that passive remote monitoring technology was particularly useful within the COVID-19 context. Our results provide important insights into the ways the older adults and their caregivers have been affected during the COVID-19 context and how to better support them in the future.

## Introduction

The COVID-19 pandemic has brought about a major upheaval in the lives of older adults and their family/friend caregivers [1] including highlighting the vulnerability of older adults living in long-term care (LTC) homes [2]. Many community-dwelling frail older adults are also at a high risk of morbidity and mortality and many have experienced a loss of services deemed nonessential that can contribute to feeling lonely and abandoned [3]. We know little about the experiences of community-dwelling frail older adults and their caregivers during the pandemic.

Our research team is engaged in a pragmatic randomized controlled trial study, “Caring Near and Far,” that is examining the effectiveness of passive remote monitoring technologies (RMT) for frail older adults receiving home care services defined as being likely to be admitted to a LTC home within the next 12 months. Passive RMT uses sensors that do not require any action by the individual for the system to work, as opposed to active RMT that requires individual participation, such as pushing a button. Information gathered from the sensors (e.g., motion sensors, cameras) is transmitted to a caregiver to alert them to a possible incident (e.g., a fall, wandering). Quantitative and qualitative data was collected from dyads of home care clients and their family/friend caregivers at three time points in the provinces of Ontario (ON) and Nova Scotia (NS), Canada. For further details on our study methodology, please see the published protocol [4].

The purpose of this study is to identify the health service experiences and preferences of frail home care clients and their family and friend caregivers during the COVID-19 pandemic. We address the following research questions:

1. How has the utilization of home and community-based services been affected during COVID-19 for frail older adults and their caregivers?
2. What is the impact of changed access to services during COVID-19?
3. How has COVID-19 affected decisions and preferences about LTC placement?
4. How has the utilization of passive RMT been affected by COVID-19?

## Main text

### Methods

We invited home care clients and their caregivers to respond to our COVID-19 related questions either during regularly scheduled interviews as part of our larger pragmatic randomized controlled trial or during a separate interview.

#### Data collection

Research coordinators and research assistants conducted the interviews by telephone. Open-ended questions added to this study focused on understanding how our study participants have been affected by COVID-19, including their utilization and preferences of health services. These questions added approximately 10 to 30 min of interview time.

#### Setting and recruitment

A total of 29 participants volunteered to respond to the COVID-19 related questions from March-June 2020 including 10 home care clients and 19 caregivers. Home care clients’ dependence on their family/friend caregivers was assessed by determining their risk of requiring higher levels of care according to the Mini Mental-State Exam [5] (to determine cognitive functioning) and the HARP Independent Activities of Daily Living assessment [6] (to determine physical functioning). These assessments indicated two home care clients were at elevated risk for requiring higher levels of care due to both cognitive decline and physical decline, six were at elevated risk due to physical decline, and two exhibited neither cognitive nor physical decline to the degree that would require elevated levels of care. The participants were located in ON (n = 15) and NS (n = 14). A total of 14 people interviewed were in the study intervention arm and 15 received usual care.

#### Data analysis

The interviews were transcribed verbatim and field notes were added by the interviewers. Through adapting a framework method [7] to guide our data analysis process, we deductively organized the qualitative results according to our 4 questions and then inductively identified themes with each question utilizing thematic analysis [8]. To ensure auditability, members of our research team involved in interviewing and managing qualitative data provided insights into important findings emerging from the interviews.

### Results

See Table 1.

**Table 1:** Questions and themes

Questions	Themes
1. How has the utilization of home and community-based services been affected during COVID-19 for frail older adults and their caregivers?	Service cancellation
	Modified access with infection control practices implemented
2. What is the impact of changed access to services during COVID-19	Emotional and physical toll on home care clients
	Increased need for family caregiver support
3. How has COVID-19 affected decisions and preferences about LTC placement?	Less likely to want to go to LTC
	I will go if I have to
4. How has the utilization of passive RMT been affected by COVID-19?	Less utilization
	No impact on utilization
	Increased utilization

#### How has the utilization of home care services been affected during COVID-19 for frail older adults and their caregivers?

##### Service cancellation

For many, the impact of COVID-19 on home care services resulted in the client or caregiver cancelling services for many reasons including: different home care workers being assigned to one client; home care workers also working in nursing homes; or having a homecare worker who tested positive for COVID-19. For some, canceling home care services was a mutual decision. “They ended up cancelling themselves. But we had talked to them beforehand…. I don't think that the homecare will come back until they’re satisfied that things are safe” (NS client 212,155).

Other services in the home, such as respite were affected during COVID-19. “So we were actually the best part of two months with no help.” (NS caregiver 222,154).

##### Modified access with infection control practices implemented

Some participants continued to receive home care services during the pandemic. “I respect that they put masks on and that they wear gloves too” (ON client 113,138). Home care agencies conducted screening prior to coming into a client’s home. One participant established their own protocols to screen home-care workers coming into their home. Many participants expressed concerns about multiple home care workers coming into the home. “We have so many people coming in the house, we make sure everybody’s got masks on and they're following protocol.” (ON caregiver 123,181). Some participants had access to physicians who provided house calls or appointments over the phone. Several participants spoke about continued access to pharmacy services and safe ways to pick up orders or prescription delivery.

#### What is the impact of changed access to services during COVID-19?

##### Emotional and physical toll on home care clients

Some participants expressed that they missed their care workers and found it lonely having fewer or no home care providers in their homes. In addition, many older adult clients reported having to do more care for themselves, such as bathing, and struggled to care for themselves safely. “He had personal care that came at least once a week, and he doesn’t have that anymore. And I think he kind of misses that because … when he gets a bath, he’s more comfortable knowing that there’s someone there in case he falls” (NS caregiver 122,137).

##### Increased need for family caregiver support

The impact of reduced services often resulted in family members providing more supports. Providing additional support was very challenging for many caregivers. One caregiver reported a two-month gap in other supports in the home. Some family members chose to live with their relative receiving home care. “Our son is now staying with us. And he does work, but he comes in the basement door, strips, showers, puts his clothes in the laundry, and then upstairs. So we feel very safe with him” (NS caregiver 122,152). Caregivers who lived with the home care client in addition to working from home faced particular challenges: “Where if we were generally working, she would be taking her own medications in certain doses, preparing her lunch herself. So, it’s changed certainly that we are here so she relies on us more as well” (ON caregiver 123,165).

#### How has COVID-19 affected decisions and preferences about LTC placement?

##### Less likely to want to go to LTC

The majority of home care clients did not want to go into LTC during the pandemic. “The deaths that are being happening in these long-term care nursing homes. I think if a person is being sent to a long-term care, it’s suicide” (NS client 212,155). Most caregivers stated they would not place their family members there during the pandemic. “I’m just glad she wasn’t in a facility because I felt I would have taken her out” (NS caregiver 122,107). One caregiver wanted enhanced regulation before she would consider LTC for her husband.

##### I will go if I have to

Conversely, some participants indicated that they would consider LTC placement if their health declined. “I would probably go if I was in that condition of not looking after myself” (NS client 112,152). They recognized that not all LTC homes experienced outbreaks of COVID-19. Another participant had experience working in LTC and was not concerned if moving to one became a necessity. “I don't really have any concerns. I worked in them, so I know the system” (ON client 113,138).

#### How has the utilization of passive RMT been affected by COVID-19?

##### Less utilization

In some cases, passive RMT was used less due to family members being more physically available during COVID-19. “I’m not able to use it as much as I did because I’m not going out…prior to COVID-19, I would use the monitor, the camera more because I was out (NS caregiver 222,155).

##### No impact on utilization

In some instances, there was no impact of COVID-19 on the use of the technology. As the system is designed to work passively, many clients think very little about the technology in their homes. “With the COVID virus or without the COVID virus, it is basically the same thing because you’re able to do it without being in contact” (NS Caregiver 222,117).

##### Increased utilization

Some caregivers found that passive RMT was more helpful during COVID-19 to ensure their loved-one was safe and secure. Caregivers who did not live with the home care client were comforted by being able to use the cameras to check on their family member while not having to be physically present and potentially spreading COVID-19. Some caregivers used the camera to monitor the number of different people coming into the home to provide personal care. Another caregiver preferred to monitor her mother using the technology rather than have multiple different home care workers coming into the home. The acceptance of various forms of technologies increased during the pandemic, especially if it supported being able to remain at home.

### Discussion

Some caregivers found that passive RMT technology was particularly useful within the COVID-19 pandemic context and some clients benefited from knowing that someone was monitoring them. These functions of passive RMTs may become more valued during the pandemic as monitoring can be done without physical contact. There may also be new applications of RMT for home care agencies who could monitor clients without going into the home.

It is imperative to support family and friend caregivers who may need to provide additional care during the COVID-19 pandemic out of necessity. While identifying the need to support caregivers of older adults is well-established [9, 10], this need is more intensified in the pandemic context. Creative ways to support caregivers are needed, such as supports that can be provided remotely (e.g., online support groups).

A key concern raised by our participants was having multiple home care providers coming into their homes. The issue of care staff working across multiple LTC homes has been clearly identified to mitigate the spread of COVID-19 [11]. Prior to the COVID-19 context, the lack of consistent home care providers was identified as a concern about quality of care [12, 13]. The current pandemic may provide additional impetus for improving various aspects that could contribute to staff retention such as improving compensation, education and training, and working conditions [14, 15].

In addition to changes in services, the pandemic created a situation in which many people were even less open to considering moving to a LTC home. Most older adults prefer to live as independently as possible in their own homes in the community [16]. Our results indicate that this may be amplified during the COVID-19 pandemic context, and there may be even greater need to expedite the development of innovative housing and care options to meet the needs of frail older adults who do not wish to move to a traditional LTC home.

In conclusion, there was clearly a diversity of experiences of the participants in our study within the context of the COVID-19 pandemic. While we expected that many community-dwelling older adults would be affected by a loss or modification of home care services [3], in this study we provided additional insights into the impact of COVID-19 on the lives of home care clients and their caregivers. Many were affected drastically by the elimination or reduction of access to services. Our results highlight the vulnerability of home care clients and their caregivers during the pandemic.

## Limitations

There are limitations of the utility of passive RMT within the context of the COVID-19 pandemic as it does not address the social isolation that many older adults may be experiencing. Also, not all home care clients have equal access to high-speed internet which could create disparities for those living in urban versus rural or remote locations. We collected data in two Canadian provinces. Our results can not be transferred to other jurisdictions, especially where different home care practices have been employed during the pandemic (e.g., different ways that services have been modified).

## Data Availability

The data analysed during this study are not publicly available as the qualitative data may be identifiable. Questions about access to deidentified data should be addressed to the corresponding author.

## Abbreviations

LTC: Long-term care
NS: Nova Scotia
ON: Ontario
RMT: Remote monitoring technology
